# Binary and Hybrid Work-Condition Maps for Interactive Exploration of Ergonomic Human Arm Postures

**DOI:** 10.3389/fnbot.2020.590241

**Published:** 2021-01-08

**Authors:** Luka Peternel, Daniel Tofte Schøn, Cheng Fang

**Affiliations:** ^1^Delft Haptics Lab, Department of Cognitive Robotics, Delft University of Technology, Delft, Netherlands; ^2^SDU Robotics, The Maersk Mc-Kinney Moller Institute, University of Southern Denmark, Odense, Denmark

**Keywords:** Work-Condition Map, ergonomic human arm posture, biomechanical model, graphical user interface, interactive exploration

## Abstract

Ergonomics of human workers is one of the key elements in design and evaluation of production processes. Human ergonomics have a major impact on productivity as well as chronic health risks incurred by inappropriate working postures and conditions. In this paper we propose a novel method for estimating and communicating the ergonomic work condition called *Binary Work-Condition Map*, which provides a visualized feedback about work conditions of different configurations of an arm. The map is of binary nature and is derived by imposing the desired thresholds on considered ergonomic and safety related criteria. Therefore, the suggested arm postures in the map guarantee that all considered criteria are satisfied. This eliminates the ambiguity compared to state-of-the-art maps that uses continuous scales derived from weighted sum of multiple ergonomics criteria. In addition, to combine the advantages of both the binary map and the continuous map, we additionally propose a *Hybrid Work-Condition Map* that rules out unsuitable workspace with the binary map approach and renders the suitable workspace with the continuous map approach. The proposed approach was tested in simulation for various tasks and conditions. In addition, we conducted subjective evaluation experiments to compare the proposed methods with the state-of-the art method regarding the usability. The results indicated that the binary map is simpler to use, while the hybrid map is a good tradeoff between the binary and the continuous map. In selecting the map, strong points of each map should be considered with respect to the requirements of a specific application and task.

## 1. Introduction

Robots have successfully supplemented human workers in modern manufacturing processes. Nevertheless, in many cases, robots did not replace the human workers, who are still an essential element at various production stages. While robots can work safely and efficiently without getting tired for extended periods of time, human workers are prone to productivity degradation when ergonomics is not taken into account. This is true both when the humans work on their own and when they work with machines, such as collaborative robots.

One of the major issues regarding human ergonomics are improper working postures, which can produce excessive joint torques that are detrimental to the current task efficiency, as well as to health and safety of the human in the long run (Keyserling and Chaffin, 1986; Kumar, 2001). Earlier methods of evaluating ergonomics of working postures, like *Rapid Upper Limb Assessment* (RULA) (McAtamney and Corlett, 1993) and *rapid entire body assessment* (REBA) (Hignett and McAtamney, 2000), used predefined heuristic tables that indicate a score of particular joint configuration. The combination of scores for all joints then gives the final score for the entire working configuration of an arm or a body. Recently, RULA and REBA have been applied to determine ergonomic postures in human-robot collaboration (Busch et al., 2017; Marin et al., 2018; Shafti et al., 2019). Other methods (Snook and Ciriello, 1991; Waters et al., 1993) used tables or equations to provide physical limits that should not be exceeded in terms of load during lifting tasks. Nevertheless, tables are more difficult to be personalized for a specific worker, and are more difficult to be generalized for different tasks and conditions. In addition, there are other important underlying indicators that affect the human ergonomics beyond kinematic posture and lifting load.

More recent methods included other indicators to optimize human working configuration, such as muscle comfort (Chen et al., 2018), physical fatigue (Maurice et al., 2016; Peternel et al., 2018b), energy consumption (Kim et al., 2010; Maurice et al., 2016), and arm manipulability (Jacquier-Bret et al., 2012; Gopinathan et al., 2017; Peternel et al., 2017; Petrič et al., 2019). In addition, they used personalized human body models (Maurice et al., 2014; Maurice et al., 2016; Kim et al., 2018a), which can be more easily integrated into collaborative robot controllers and generalized for many tasks. When a human is collaborating with a robot, we can use the robot to optimize the collaborative task execution based on the dynamical models of the human worker. Methods in Vahrenkamp et al. (2016)and Peternel et al. (2017) allowed the robot to plan the optimal handover of tools and objects between humans and robots by considering various factors, such as human dexterity and joint torques. Methods in Kim et al. (2018a) and Peternel et al. (2017) enabled the robot to detect the overloading joint torques in human body and then physically guide the human to change configuration online during the working process. Other methods in Peternel et al. (2018b, 2019) let the robot to estimate the human worker's muscle fatigue and then minimized it by reconfiguration of task execution. A similar method was also employed for ergonomic reconfiguration of human operator's arm in teleoperation (Peternel et al., 2020).

In Mansfeld et al. (2018), the authors proposed a concept called *Safety Map*, which used the information about robot inertia in different states of the workspace in combination with human injury data, to give workers a visual representation about the safety of interaction. Nevertheless, this map only examined safety in terms of possible collisions and gave no consideration to other major factors that affect the human ergonomics, such as joint torque, posture, and fatigue. Several methods used either one of these factors as ergonomics criterion (Kim et al., 2018a,b; Lorenzini et al., 2019; Peternel et al., 2019; Petrič et al., 2019). The method in Maurice et al. (2016) considered multiple criteria, but did not provide a combined overall ergonomics index. The methods in Peternel et al. (2017) and Chen et al. (2018) combined two or more criteria to derive the optimal arm posture, but lacked a visual interface to convey the information about ergonomic suitability of the whole workspace.

In Vahrenkamp et al. (2016), the authors proposed a concept called *Interaction Workspace*, which provided a visual color map of the workspace that indicated what arm postures are most suitable for task execution. Each posture had an index value that depended on a combination of several ergonomics criteria, such as human joint torque and dexterity. The index values were represented by color spectrum (i.e., one side of color spectrum for unfavorable values and the other side for favorable values). Nevertheless, the overall index for each posture was calculated by a weighted sum of all involved criteria (Vahrenkamp et al., 2016), therefore the contribution of each individual criterion may be unclear to users. Specifically, it may not be intuitive to a casual worker (and even experts) what a specific overall index value and its assigned color mean in terms of individual ergonomics factors. Moreover, due to the weighted sum, the overall index cannot guarantee that a given working posture does not exceed ergonomic thresholds of any individual criteria. These problems are also shared with RULA (McAtamney and Corlett, 1993) and REBA (Hignett and McAtamney, 2000), which provide a combined score from individual scores of different joints.

To resolve the above-mentioned issue, we propose a novel concept called *Binary Work-Condition Map*. Unlike methods that use weighted sum of various criteria (Vahrenkamp et al., 2016), the proposed method uses a threshold based approach for various criteria to obtain the overall ergonomics index at different positions of the workspace. This index is therefore binary and can potentially be more intuitive and easier to understand. For example if the index is one (logical true) in a given position, it means that all ergonomics criteria comply with the respective thresholds, which can be defined by the established safety and health standards and set by experts. If it is zero (logical false), then it is clear to a casual worker that a given working position does not satisfy all the standards and thresholds set by experts. In multi-color map (Vahrenkamp et al., 2016), this is not clear, because even in the safest "green" area, one of the thresholds might be exceeded, if the other factors are predominately satisfied due to the weighted-sum nature of derivation.

An additional contribution of the proposed work-condition map method is a novel display feature that can indicate ergonomic states of multiple arm postures sharing the same endpoint position for human arms, which possess such intrinsic kinematic redundancy. Such a feature is missing in the state-of-the-art work-condition map methods.

## 2. Methods

**Figure 1:**
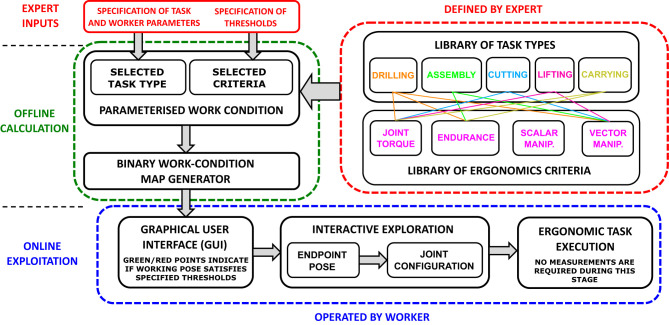
An overall diagram of the generation and usage of the proposed interactive task-oriented Binary Work-Condition Map.

An interactive Binary Work-Condition Map guides human workers to place their arms in appropriate postures for performing quasi-static manipulation tasks in an ergonomic and safe way. The method takes into account multiple task-related parameters and upper limb dynamic model (Saul et al., 2015) to create and if necessary update the binary map of suitable and non-suitable working postures. This map can be used to provide human workers with posture guidance for accomplishing tasks independently. Alternatively, it can be shared with collaborative robots that are working together with human co-workers in order to optimize the collaboration. The workflow framework of the proposed interactive Binary Work-Condition Map is shown in Figure 1.

When a human worker is assigned to perform a specific task within the manufacturing process, the method categorizes the task by its type. The type of the task is associated with either a single criterion or multiple ergonomics criteria, which are used to evaluate various ergonomics and safety related work conditions during manipulation (e.g., joint torque, posture, fatigue, etc.). Different task types have different sets of associated criteria (see the top-right corner of Figure 1) and are represented in a library that is predefined based on the task knowledge provided by experts. For instance, in a task that requires a large force production for a short duration (e.g., lifting an object), the key criteria are joint torque and posture due to the large force, while fatigue is not dominant due to the short duration. In a task that requires a small force production for a long duration, fatigue becomes dominant and instantaneous joint torques play a lesser role.

The major types of criteria that we considered are joint torque, endurance, and manipulability. Instantaneous *joint torque* is important in terms of safety, as it can lead to various short-term and long-term injuries (Keyserling and Chaffin, 1986; Kumar, 2001; Kim et al., 2018a). On the other hand, integrated joint torque over time will lead to muscle fatigue (Peternel et al., 2018a,b, 2019), which can degrade the human worker's performance and endurance (De Luca, 1984; Enoka and Duchateau, 2008; Ma et al., 2009). The fatigue therefore translates to *endurance time*, after which the worker cannot perform the required task production forces. The posture affects the manipulability of the human arm, which defines how well it can produces motion or forces at the endpoint (i.e., hand) in various directions of Cartesian space (Yoshikawa, 1985a,b; Petrič et al., 2019). The manipulability measure can be either scalar or vector. *Scalar manipulability* measure indicates how well the arm endpoint can produce both motion and force in all directions. For example, this measure is useful when the worker has to perform complex assembly tasks, where both motion and force are important in various directions to complete them. On the other hand, *vector manipulability* measure indicates how easy the arm endpoint can produce either motion or force in a specific direction of Cartesian space. For example, this measure is useful when the worker has to lift or carry a heavy object, where a good force production capability is necessary in the direction of the gravity.

Table 1 shows a library of considered tasks and criteria. For this study we considered five common manipulation tasks and four ergonomics criteria that are most relevant for manipulation tasks. Drilling task usually requires holding a heavy tool and producing relatively large forces for a prolonged time, therefore joint torque and endurance time are critical. Furthermore, the drilling force is in a specific direction, thus vector manipulability should be considered. Cutting and lifting require large effort as well, but only for a shorter time compared to drilling, therefore endurance time is not as important. On the other hand, carrying is typically a longer action than lifting and therefore requires endurance time consideration. Finally, a typical part assembly might not demand a lot of joint torque effort, however it may take a while thus endurance time is important. In addition, complex assembly tasks require producing forces and motion in various directions, therefore scalar manipulability is more important than vector manipulability. Note that this is a general framework and the considered tasks and criteria can be expanded. Certain criteria may become relevant or irrelevant depending on the specifics. For example, if an assembly task takes a lot of time, endurance time is relevant, while if it can be done quickly, it becomes irrelevant. When new tasks are added, a conceptual analysis is necessary to determine which ergonomics criteria are relevant.

**Table 1:** A library of common manipulation tasks and ergonomics criteria that are considered in this study.

	**Joint** **torque**	**Endurance** **time**	**Scalar** **manipulability**	**Vector** **manipulability**
Drilling	✔	✔	✗	✔
Assembly	✗	✔	✔	✗
Cutting	✔	✗	✗	✔
Lifting	✔	✗	✗	✔
Carrying	✔	✔	✗	✗

*The marks ✔ and ✗ indicate whether a specific criterion is relevant for a specific task or not, respectively*.

When all the relevant criteria are determined according to the associated task type, the experts can set the thresholds for each ergonomics criteria. For example, if a certain amount torque is known to cause injuries and long-term health problems, the threshold is set conservatively below such limit. We should stress again that thresholding approach is the key difference compared to the existing methods that use weighted sum of criteria (Vahrenkamp et al., 2016). The proposed approach can guarantee that the preset thresholds for individual criteria will not be exceeded when the worker maintain the posture in the prescribed ergonomic area, while the continuous-condition maps obtained by a weighted sum cannot guarantee that.

The threshold can be set as a fixed limit below or above which the working arm posture is ergonomic as

(1) $$\begin{array}{l} c_{i}=\left\{ \begin{array}{cc} 1 & \text{ if }v_{i}\left(\text{q},\text{ P}\right)<v_{th,i}\;\\ 0 & \text{ if }v_{i}\left(\text{q},\text{ P}\right)\geq v_{th,i} \end{array}\right., \end{array}$$

where *c*_*i*_ is the binary index of *i*-th criterion, *v*_*i*_ is the *i*-th variable (e.g., joint torque, endurance time, etc.), and *v*_*th, i*_ is the respective threshold. Variable *v*_*i*_ depends on arm posture that is defined by joint angles **q** and input parameters **P**, which include task production force **f**_*ref*_ and other conditions. Note that inequality signs in (1) can be reversed, depending whether the more ergonomic state is below or above threshold. For example, in case of joint torque, the more ergonomic state is naturally below the threshold torque. In case of scalar manipulability, it is above the threshold since the larger manipulability is more ergonomic.

Alternatively, the threshold can be set as a range when the variable should be within some interval as

(2) $$\begin{array}{l} c_{i}=\left\{ \begin{array}{cc} 1 & \text{ if }v_{min,i}<v_{i}\left(\text{q},\text{ P}\right)<v_{max,i}\\ 0 & \text{ if }\mathrm{else} \end{array}\right., \end{array}$$

where *v*_*min, i*_ and *v*_*max, i*_ are minimum and maximum threshold of the range, respectively. For example, (2) can be used instead of (1) when we want to make sure the joint torque does not exceed the safe limits (upper threshold *v*_*max*_), but on the other hand, we do not want the worker to become too inactive (lower threshold *v*_*min*_).

The parameters are passed on to the Binary Work-Condition Map generator that creates a workspace map for a given task by calculating a binary ergonomic state for each arm posture within the workspace as

(3) $$\begin{array}{l} e_{k}^{bin}\left({\text{q}}_{k}\right)=c_{1}\wedge c_{2}\wedge ...c_{n}, \end{array}$$

where $e_{k}^{bin}$ is the combined overall binary index for *k*-th human arm configuration **q**_*k*_, calculated by a logical AND operation ∧ among the individual binary indices of various criteria *c*_*i*_, *i* = (1, 2, ...*n*). The considered criteria (joint torque, endurance time, and scalar and vector manipulability) and Binary Work-Condition Map generator are defined and described in the following subsections.

For comparison, the proposed threshold based approach is in contrast to the weighted-sum based approach in (Vahrenkamp et al., 2016), which produces a continuous ergonomic state for each arm posture as

(4) $$\begin{array}{l} e_{k}^{con}\left({\text{q}}_{k}\right)=c_{1}w_{1}+c_{2}w_{2}+...\;c_{n}w_{n}, \end{array}$$

where in this case criteria *c*_*i*_ have continuous values and *w*_*i*_ are their respective weights.

The advantage of the binary map is to be able to guarantee that the thresholds are met for all ergonomics criteria, however it has only binary states and better configurations among the good ones cannot be distinguished. On the other hand, the continuous map has more states and can therefore distinguish between different levels of good configurations, however it cannot guarantee that the thresholds for all criteria are met, even if the configuration is in the green section. That is because weighted-sum approach may produce a high score when majority of criteria are high, while one of them is very low and below a threshold.

In order to exploit the advantages of both the binary map and the continuous map, we also propose a novel hybrid map. In this approach, the binary map is used as a mask over the continuous map in order to filter out all configurations that do not meet the thresholds of all criteria. The remaining suitable sections of workspace are then colored by the continuous map in order to provide the user with a distinction between different levels of good configurations. The proposed hybrid map can be mathematically formalized as

(5) $$\begin{array}{l} e_{k}^{hyb}\left({\text{q}}_{k}\right)=e_{k}^{bin}\left({\text{q}}_{k}\right)\cdot e_{k}^{con}\left({\text{q}}_{k}\right), \end{array}$$

In practice, the section of workspace that is red in the binary map remains red, while the green section can be recolored with a color scale to indicate multiple levels of goodness. Note that if applying very strict thresholds in the binary map, the border of the mask might be already in the green sections of the continuous map. To exploit the full color spectrum and better to distinguish different levels of goodness for different points, the continuous sections of the hybrid map can be rescaled; for example, so that yellow color will start at the border instead of green color.

### 2.1. Joint Torque

The quasi-static relation between the human arm joint torques and endpoint force related to the task production is defined as

(6) $$\begin{array}{l} \tau ={\text{J}}^{T}\left(\text{q}\right)\text{f}+\text{g}\left(\text{q}\right), \end{array}$$

where **τ** is the joint torque vector of the human arm, **f** is the endpoint force vector, **J** is the geometric Jacobian matrix of the human arm, **q**_*h*_ is the joint angle vector, and **g**_*h*_ is the gravity torque vector of the arm. As it can be seen from (6), the joint torque is affected by the force that is actively produced as a result of task performance, and the gravity of the human arm itself. The joint torque is calculated by using a human arm biomechanical model from Fang et al. (2018), which is based on the model developed in Holzbaur et al. (2005).

### 2.2. Endurance Time

Endurance time in which the human can perform the task with a specified force **f** is related to physical fatigue. We estimated the fatigue based on the model proposed in (Peternel et al., 2018b), which follows first-order system dynamics as other established models from the literature (Ma et al., 2009). Here we used joint torque as an effort estimation parameter as in (Maurice et al., 2016; Lamon et al., 2019). The fatigue model for each joint is defined as a first-order system of differential equations as

(7) $$\begin{array}{l} \frac{du_{i}\left(t\right)}{dt}=\left\{ \begin{array}{cc} \left(1-u_{i}\left(t\right)\right)\frac{\left|\tau_{i}\left(\text{q},t\right)\right|}{\lambda_{i}} & \text{if }|\tau_{i}\left(t\right)|\geq \tau_{th,i}\\ -u_{i}\left(t\right)\frac{R}{\lambda_{i}} & \text{if }|\tau_{i}\left(t\right)|<\tau_{th,i} \end{array}\right., \end{array}$$

where *u*_*i*_ ∈ [01] is the *i*-th joint fatigue index, τ_*i*_ is calculated from (6) for a given time *t* and configuration **q**, λ_*i*_ is a capacity parameter that determines the joint-specific fatigue characteristics. The parameters λ are dependent on individual human and joint. The higher the λ is, the more effort τ over time it takes for the fatigue to occur. The parameter *R* is a recovery rate, which determines the speed of fatigue reduction after the arm is relaxed. In our experiments we used a conservative value *R* = 0.5, as in (Peternel et al., 2018b) for all the joints. Other recovery rates can be found in literature (Ma et al., 2010). We used the threshold τ_*th, i*_ to determine when the arm joint is relaxed. When the joint torque is larger than this threshold, the model is in fatigue increasing mode, otherwise it is in recovery mode.

The values of fatigue capacity parameters λ of individual arm joints can be estimated by the method proposed in (Peternel et al., 2018b). In this procedure, the human produces several reference joint torques τ_*calib*_ for the amount of time *T*_*calib*_, after which the human cannot endure it anymore or feels uncomfortable. In other words, one chooses τ_*calib*_ and measures respective *T*_*calib*_. Capacity λ for each reference torque τ_*calib*_ is then derived by

(8) $$\begin{array}{l} \lambda =-\frac{|\tau_{calib}|\cdot T_{calib}}{\ln\left(1-0.993\right)}, \end{array}$$

where the full capacity is assumed to be reached after five time constants, i.e., *u* = 0.993. The mean value of λ parameters, calculated by (8) for different reference forces, is then used as the final estimation of fatigue capacity for each joint separately.

The maximum endurance time *T* for an arbitrary joint torque τ is then obtained by,

(9) $$\begin{array}{l} T=-\frac{\lambda \cdot \ln\left(1-0.993\right)}{\left|\tau \right|}. \end{array}$$

### 2.3. Scalar Manipulability

The scalar manipulability measures how well the arm endpoint can produce both motion and force in all direction of Cartesian space, and can be derived as Yoshikawa (1985b)

(10) $$\begin{array}{l} w=\sqrt{\det\left(\text{J}\left(\text{q}\right)\text{J}{\left(\text{q}\right)}^{T}\right)}, \end{array}$$

where the higher value means more capacity to produce both motion and force at the endpoint. If the task requires complex manipulation that involves movements and force of the endpoint in various directions (e.g., complex assembly), it should be performed around the configuration where the manipulability *w* is the highest.

### 2.4. Vector Manipulability

The manipulability can also be examined on a vector level by using Eigen decomposition or singular value decomposing of arm Jacobian matrix (Yoshikawa, 1985b). Velocity manipulability is derived as

(11) $$\begin{array}{l} \text{U}\Sigma {\text{V}}^{T}=\text{J}\left(\text{q}\right)\text{J}{\left(\text{q}\right)}^{T}, \end{array}$$

where **Σ** are singular values, while **U** and **V** are left and right singular vectors, respectively. **Σ** and **U** determine the size and shape of velocity manipulability ellipsoid, respectively. The size of a vector from the center of the ellipsoid to its surface in any direction tells how well the arm endpoint can move in that direction. Force manipulability is derived as

(12) $$\begin{array}{l} \text{U}\Sigma {\text{V}}^{T}=(\text{J}\left(\text{q}\right)\text{J}{\left(\text{q}\right)}^{T}{)}^{-1}, \end{array}$$

where singular values and vectors have similar roles as in velocity manipulability. The force manipulability ellipsoid is able to tell how well the arm endpoint can produce or sustain forces in a certain direction.

The major axes of force and velocity manipulability are orthogonal; therefore if the arm in a given configuration can produce large velocities in a certain direction, then a large force cannot be produced in that direction, and vice-versa. For example, if the task requires to produce or sustain high forces in a certain direction (i.e., lifting a heavy object), the highest force manipulability vector should be aligned with that direction [i.e., (12) should be used]. If we need to move the manipulated object fast in a certain direction, the highest velocity manipulability vector can be aligned with that direction [i.e., (11) should be used]. In connection to the scalar manipulability from (10), high *w* tends to make velocity and force manipulability ellipsoids closer to a sphere.

### 2.5. Binary Work-Condition Map Generator

To evaluate all possible arm postures, subject to selected workspace and joint limits, the whole configuration space of human arm is discretized in terms of the *Cartesian-Posture-Swivel-Angle* (CPSA) representation of human arm configuration (Fang et al., 2019). In this CPSA representation, a human arm configuration is expressed by a 3-degrees-of-freedom (DoF) position and 3-DoF orientation of human hand, plus 1-DoF swivel angle of the elbow, which is determined by the angle between a shoulder-elbow-wrist human arm plane and a vertical plane (Tolani et al., 2000).

**Figure 2:**
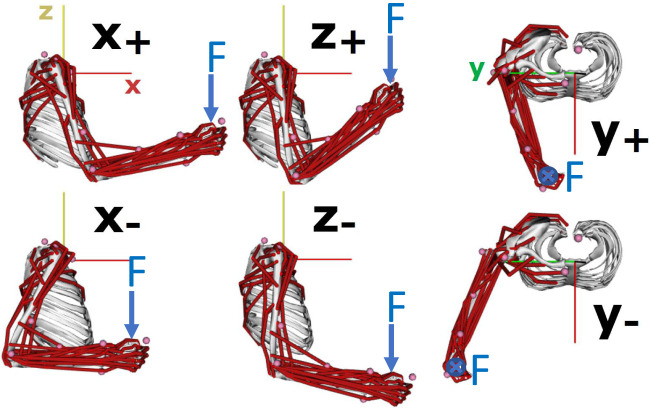
Discretization of arm posture in terms of hand position in the proposed Binary Work-Condition Map by using human biomechanical model.

**Figure 3:**
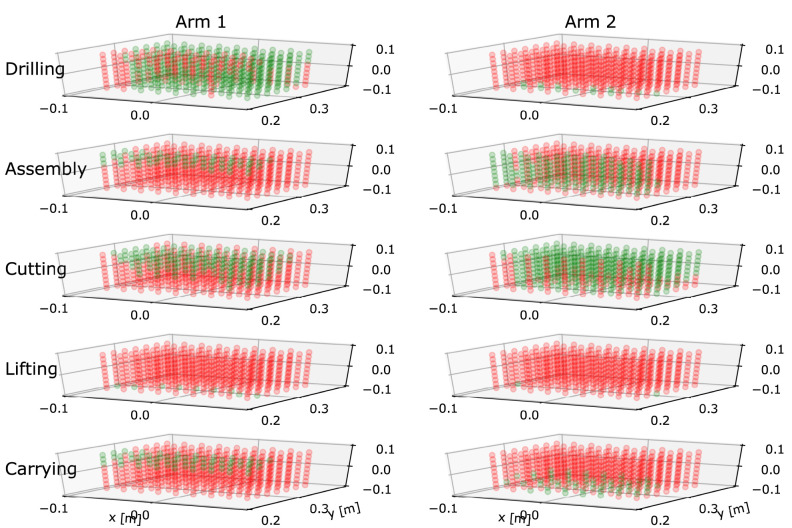
A series of Binary Work-Condition Maps generated for five different tasks and two different human arm dimensions. Arm 1 had upper arm and forearm lengths 0.250 and 0.266 m, repetitively. Arm 2 had upper arm and forearm lengths 0.323 and 0.232 m, repetitively. X-axis point right of the body, y-axis points in front of the body and z-axis point vertically. The frame origin is at the right shoulder position. Green colored point indicates that if the endpoint is placed in it, at least one arm configuration exist where all criteria are within thresholds. Red colored point indicates that there are no ergonomic configurations in that position.

Figure 2 provides an example of the discretization of arm posture in terms of hand (endpoint) position and Figure 3 provides two examples of the discretization of the arm posture in terms of swivel angle. A hand position and swivel angle step sizes are predefined in advance. Every possible arm posture is tested under the desired external force required to produce the task (e.g., *F* shown in Figure 2). This is done automatically through an individual simulation using an OpenSim biomechanical model (Saul et al., 2015; Seth et al., 2018). The collected data for each posture is analyzed by (3) according to all the associated criteria and predefined thresholds. If all the criteria are satisfied and *e*_*k*_(**q**_*k*_) = 1, the arm posture **q**_*k*_ will be labeled as a feasible configuration, otherwise it will be labeled as an infeasible posture.

After the offline calculation stage, the tested hand positions are visually presented to a worker through the developed graphical user interface (GUI) as a binary map of Cartesian points, where ergonomically feasible points are displayed by green color and infeasible by red color. Prior to the interactive exploration stage, the human worker attaches a set of pose-measurement markers on the anatomical landmarks of his/her arm. During the interactive exploration stage, the current arm posture is then captured by an optical motion capture system and reconstructed in a graphical user interface (GUI) in real time (Fang et al., 2018). This enables the worker to interactively explore the workspace in real time through the GUI and generated map in the online exploitation stage.

Since there is a redundancy in the human arm, there are multiple possible configurations for a single hand position. The hand position is displayed as feasible (green color), if there is at least one feasible configuration within that hand position. The worker can then move the hand into that point and explore it by changing the configuration through swivel angle and redundant DoF in real time. If the configuration satisfies the criteria, the elbow point of the simulated human arm on the interactive map turns green, if not it turns red. Note that redundancy was not considered in (Vahrenkamp et al., 2016), therefore the proposed redundancy-display approach is novel in terms of interactive maps.

The examples of maps created for two arms of different dimensions are illustrated in Figure 3. Through interactive self-supervised exploration manner, the worker is able to establish an intuitive sense of how he/she should place the arm in appropriate configurations for performing the specified task. This self-supervised exploration can be further divided into practicing in the air and practicing with the real tool to help the user memorize the desirable arm configuration step by step. When a feasible arm posture is selected and memorized after the interactive exploration, the worker can execute the actual task without the assistance of the GUI and motion capture system.

## 3. Evaluation and Results

The evaluation was separated into concept demonstration and experiments. The concept demonstration included demonstration of all aspects that does not include subjective factors of human worker (i.e., parts outside of blue area in Figure 1). These included technical calculation of work-condition maps taking a combination of different task parameters and criteria into account. The additional experiments then evaluated aspects that involve subjective perception of human worker, i.e., usability factors of the developed binary and hybrid maps compared to the existing continuous map.

### 3.1. Concept Demonstration

To evaluate the offline part of the method, we calculated a Binary Work-Condition Map for each of the five considered tasks from the library in Table 1. For each map, the associated relevant ergonomics criteria were used to determine whether the available arm postures within the workspace are suitable or not. A 0.16 x 0.12 x 0.16 m cuboid in front of the body was selected as the workspace, with its center at (*x, y, z*) = (0.0, 0.3, 0.0) m with respect to a base frame located at the right shoulder center. The positive x-axis of the base frame points rightwards from the shoulder, while the positive y-axis and z-axis points forwards and upwards, respectively. The condition was calculated for every point within the cuboid with resolution of 0.02 m. Note that the workspace and resolution can be adjusted depending on the scenario.

To demonstrate the effect of different arm dimensions on the calculation of the map, we generated five maps for two right arms of different dimensions. For the first human, the arm upper arm length was 0.250 m and the forearm length was 0.266 m. For the second human, the arm upper arm length was 0.323 m and the forearm length was 0.232 m. We used one average and one extreme arm dimensions in order to highlight the conceptual differences.

Ten maps are produced based on the calculated results shown in Figure 3. By observing the maps on the figure, we can see that tasks have major influence on the map layout. For instance, the more demanding tasks in terms of physical effort, e.g., lifting and carrying (fourth and fifth rows), have very few arm configurations in green state that satisfy all selected ergonomics criteria. On the other hand, tasks that require less physical effort, like cutting (third row), have more arm configurations in green state. While complex assembly (second) is not a physically demanding task, it does have requirements from high manipulability; therefore areas, where the arm has to be extended, are in red state. Note that in order to highlight the differences between the tasks, we intentionally used relatively strict thresholds.

The influence of arm dimensions is also clearly visible by comparing the two columns. Different arm dimensions produced noticeably different values of scalar manipulability for assembly task (second row), and different values of velocity manipulability for the cutting task (third row). In addition, the maps for the drilling task were also considerably different because of a different combination of force manipulability and joint torque results due to different arm dimensions.

**Figure 4:**
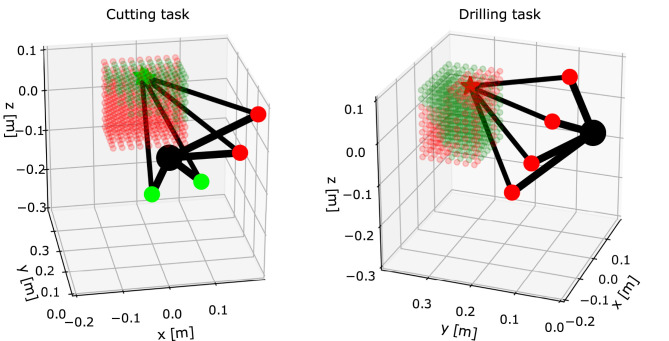
A closer look at two of the maps from Figure 3 with the same human arm displayed in different configurations. The examples show the arm in the same endpoint position (*x, y, z*) = (0.0, 0.28, 0.08) m, with four different configurations, for two different tasks. The shoulder is located at (*x, y, z*) = (0, 0, 0) m and is displayed by a large black sphere. The elbow is displayed by either green or red sphere, which indicates whether the configuration satisfies the ergonomics criteria. Hand (endpoint) is displayed by either green or red star, where there is at least one ergonomics configuration when it is colored in green.

Figure 4 shows a closer look at two maps of the same human arm for two different tasks from Figure 3. Four arm configurations of evenly spaced swivel angles with the same endpoint position are displayed on each map (the resolution of swivel angle is 30 degrees). Since the work conditions are different because of the different tasks, the ergonomic states are different. In the cutting task (left plot) the endpoint position is in a green state, since there are two out of four configurations that satisfy all ergonomics criteria. Whether the configuration is ergonomic or not is indicated by green or red elbow, respectively. On the other hand, in the drilling task (right plot) the same endpoint position is in a red state, since there are no configurations that satisfy all ergonomics criteria. Therefore, all four configurations have red elbow.

Note that in this example we used four configurations for every endpoint position within the selected workspace. The amount of configurations per endpoint can be arbitrarily increased or decreased, depending on the use cases.

### 3.2. Experiments

The conceptual differences and advantages of the binary map compared to the continuous map were highlighted in section 2, and the main features of the proposed method were shown in section 3.1. Additionally, we performed experiments to compare the different types of maps in terms of usability factors. The goal of the experiment was to evaluate subjective aspects of the proposed binary and hybrid-condition maps, compared to the continuous-condition map. We chose the continuous map as a benchmark in the comparison since it is a state-of-the-art method. Unlike the proposed method, the continuous map method (Vahrenkamp et al., 2016) did not consider redundant DoF of human arm and did not have any visualization solution for the redundant DoF. Therefore, in order to make a fair comparison, the experiments were performed using degenerate maps constrained on a 2D vertical plane, which is parallel to the human body sagittal plane and passes through the shoulder center.

We used 15 male participants in the experiment with age 27.60 ± 8.88 years, upper arm length 32.23 ± 1.85 cm and forearm length 27.93 ± 1.30 cm. The participants were briefed about the experiment procedure and the purpose of the experiment, and gave an informed consent to participation. We adapted the biomechanical model and parameters based on each individual participant during the calibration stage prior to the experiment.

**Figure 5:**
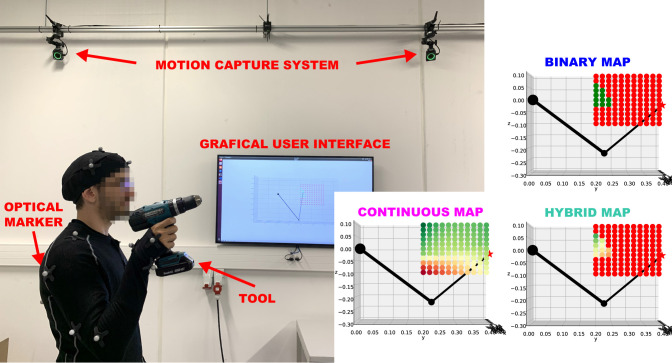
Experiment setup consists of a motion capture system (Optitrack) for tracking the human posture in real time and a graphical user interface (GUI) on a monitor that provides on-line guidance for locating ergonomic arm postures to a human worker. Examples of binary, continuous and hybrid maps rendered by the GUI are shown on the right side. Note that the difference between the maps arises from two completely different underlying concept of calculating the maps. The binary map uses thresholding and binary AND operation between different criteria [i.e., (3)]. The continuous map uses weighted sum of those criteria [i.e., (4)]. The hybrid map uses a combined approach [i.e., (5)].

The experiment setup (see Figure 5) included a motion capture system (Optitrack) that measured human arm configuration in real-time and a display (GUI) that showed the ergonomics maps with respect to the virtual copy of the human arm. The virtual copy of the arm moved in the same manner as the real arm according to the measured configuration.

Before the actual experiment, the participant conducted a familiarization experiment in order to get familiar with the setup and the methods. The experimenter explained to the participant the implications of the different colors of the map in layman terms (e.g., the green color indicates good arm working posture and red indicates bad arm working posture). During the actual experiment the participant was instructed to explore workspace and to select an arbitrary suitable arm working posture. After their selection, the participant had to then produce the actual task, i.e., holding a heavy drilling tool at the selected position for 1 min. This procedure was sequentially done for all three maps. The order of maps as they were performed in the experiments was randomized between the participants.

After the experiments, we performed the subjective evaluation by a Likert-type of questionnaire, where the participant had to report the degree of agreement with the given statements:

- *S1*: The binary map is not ambiguous to indicate a good working posture.
- *S2*: The continuous map is not ambiguous to indicate a good working posture.
- *S3*: The hybrid map is not ambiguous to indicate a good working posture.
- *S4*: I feel it took effort to place my arm in a good configuration by binary map.
- *S5*: I feel it took effort to place my arm in a good configuration by continuous map.
- *S6*: I feel it took effort to place my arm in a good configuration by hybrid map.
- *S7*: I felt comfortable with the configuration selected by the binary map.
- *S8*: I felt comfortable with the configuration selected by the continuous map.
- *S9*: I felt comfortable with the configuration selected by the hybrid map.

There were five possible levels of agreement (score is in the brackets): strongly agree (2), agree (1), neutral (0), disagree (–1), strongly disagree (–2). *S1*–*S3* evaluate the initial phase of the method, where the user has to visually search for and select a suitable configuration on a given map. *S4*–*S6* evaluate the middle phase of the method, where the user has to explore and navigate to the selected configuration. *S7*–*S9* evaluate the final phase of the method, where the user has to perform the task in the selected configuration. Additionally, we asked the participants to rank the methods according to their overall preference, where 3 points were given to the best and 1 point the worse method in terms of preference.

To check for significance of the differences between subjective scores for the three methods, we performed a statistical analysis using paired sample *t*-tests. The statistical significance was set to 0.05 and statistical power to 0.8. Power analysis indicated that sample number of 14 was sufficient under the given parameters. The datasets were checked for normality by performing the Shapiro-Wilk test. If the dataset did not pass this test, non-normal distributed data was corrected by a rank-transformation before the main test.

**Figure 6:**
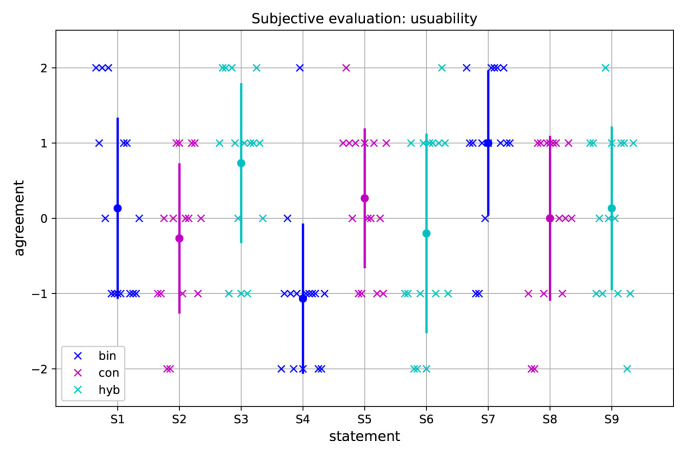
Results of subjective evaluation for usability. The statements are listed on x-axis, while the level of agreement in terms of score is shown on y-axis. Blue color is associated with the statements related to the binary map, magenta color is associated with the statements related to the continuous map and cyan color is associated with the statements related to the hybrid map. The dot represents mean score and the vertical line represents standard deviation. The individual data points are marked by crosses. In essence, positive scores for *S1*–*S3* indicate that the method was not ambiguous. On the other hand, negative scores for *S4*–*S6* indicate that the method took less effort to use. Finally, positive scores for *S7*–*S9* indicate that the task execution in the selected configuration was comfortable.

The results of the subjective evaluation for usability are shown in Figure 6. Additionally, the average degree of agreement to the statements *S1*–*S9* is shown is Table 2. On average, the participants rated the binary map less ambiguous compared to the continuous map. However, the difference was not statistically significant (*p* = 0.442). The hybrid map was also rated less ambiguous compared to the continuous map. The difference was statistically significant (*p* = 0.030).

**Table 2:** Results of subjective analysis of usability.

**Aspect**	**Statement**	**Agreement**
Not ambiguous to indicate a good working posture.	S1 (binary)	0.13 ± 1.20
	S2 (contin)	-0.27 ± 1.00
	S3 (hybrid)	0.73 ± 1.06*
It took effort to place my arm in a good configuration.	S4 (binary)	-1.07 ± 1.00*
	S5 (contin)	0.27 ± 0.93
	S6 (hybrid)	–0.20 ± 1.33
Comfortable with the selected configuration.	S7 (binary)	1.00 ± 0.97*
	S8 (contin)	0.00 ± 1.10
	S9 (hybrid)	0.13 ± 1.09

The values represent the mean degree of agreement to the statements S1–S9 and respective standard deviation. Positive value indicates agreement, while negative indicates disagreement with the statement for a given map. Symbol * indicates whether there is a significant difference with respect to the continuous map (benchmark).

On average, the participants felt that by using the continuous map it took much more effort to explore and navigate to the selected configuration compared to the binary map. The difference was statistically significant (*p* = 0.006). The same was true for the hybrid map when compared to the continuous map. However, the difference was not statistically significant (*p* = 0.389).

On average, the participants felt that performing the task in the selected configuration, by using the binary, was more comfortable compared to the one selected by the continuous map. The difference was statistically significant (*p* = 0.038). The same was true for the hybrid map when compared to the continuous map. However, the difference was statistically not significant (*p* = 0.784).

**Figure 7:**
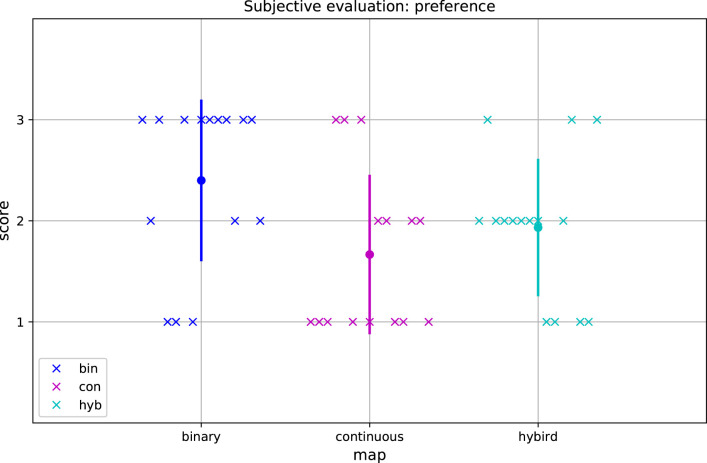
Results of subjective evaluation for preference. The maps are listed on x-axis, while the preference score is shown on y-axis. Blue color is associated with the statements related to the binary map, magenta color is associated with the statements related to the continuous map and cyan color is associated with the statements related to the hybrid map. The dot represents mean score and the vertical line represents standard deviation. The individual data points are marked by crosses. Score 3 is the best and score 1 is the worse in terms of preference.

The results of the subjective evaluation for overall preference of maps are shown in Figure 7. The participants generally preferred the binary map to either the hybrid map or the continuous map, and most of them gave the binary map the highest score. However, the preference difference between the binary map and the continuous map was statistically not significant (*p* = 0.077). Neither was significant the difference between the hybrid map and the continuous (*p* = 0.433).

## 4. Discussion

The main strength and advantage of the binary map compared to the continuous map is that it can guarantee the thresholds for all criteria are satisfied. This is not the case with the continuous map, since it uses weighted sum to derive the overall index for a given position. For example, the continuous map does not guarantee that all criteria are within the ergonomic thresholds, even in the high-value points (green color). This is a conceptual advantage which was highlighted in the section 2. The hybrid map uses the binary map concept to rule out the configurations that do not satisfy all the thresholds; therefore it exploits the main conceptual advantage of the binary map. On the other hand, it uses continuous map concept to indicate the different levels of ergonomy among the suitable ones in order to increase the resolution for the user.

The results of experiments and subjective evaluation showed that the participants found the binary map and the hybrid map less ambiguous, compared to the continuous map. This can be mostly likely attributed to the binary nature of the map, since the map gives two distinct states and therefore it is clear to the user whether the posture is either ergonomic or not (i.e., whether the thresholds set by the expert are satisfied or not). On the other hand, the continuous map has multiple states and gives a range of ergonomic values, which can be ambiguous. Medium value (yellow color) in the continuous map can be achieved by different combinations of criteria conditions, for example: non-ergonomic torque and ergonomic manipulability, or ergonomic torque and non-ergonomic manipulability, or borderline ergonomic for both torque and manipulability. Just by looking at the map, it is impossible even for an expert to know for sure which combination produced the given color, let alone a casual worker. However, the hybrid map was rated even less ambiguous which might be attributed to the exploitation of advantages of both the binary and the continuous map; a clear division between unsuitable and suitable configurations areas, but a continuous pattern within the suitable ones that permits more resolution in the selection of the best among the good ones. Therefore, this hints that the hybrid map and the binary map might be more suitable for casual workers that are not experts in ergonomics.

The results of subjective evaluation also showed that the participants felt it took less effort to use the binary map to explore and navigate to the selected configuration, compared to the continuous map. This could potentially be attributed to the binary map concept that rules out a considerable number of configurations for not satisfying all the thresholds. The continuous map concept has continuous states across all the workspace and therefore more options to navigate through. In addition, more continuous states can take more attention from the user in order to distinguish between the different tones of color while exploring through the map. As the results showed, the difference in perceived effort between hybrid map and the continuous map is not as large, which can be attributed to hybrid map taking aspects from both the binary map and the continuous map. Based on this, we recommend using the binary map when new working configurations are changing quickly in order to minimize the perceived workload on the user. If that is not the case, we recommend using the hybrid map in order to exploit also the advantage of the continuous map.

The perceived better comfort in the configurations selected by the binary map can be attributed to the binary map guaranteeing that all ergonomic thresholds are being met through the underlying thresholding approach. On the other hand, the continuous map uses weighted-sum approach and does not guarantee that all ergonomic thresholds are satisfied, even for highly rated configurations. We recommend using the binary map when satisfying thresholds for all criteria is of primary importance.

Surprisingly, the participants did not perceive the same comfort difference for the hybrid map. Different participants might subjectively weigh different relevant criteria in different ways (not equally), however equal weights among different criteria were assumed in the experiments for the hybrid and continuous maps. By using the binary map, the participants could choose the configuration, which they felt it is the most ergonomic, among several options in the green area. This implies that they might have potentially used their "embedded" non-equal weights to explore and search for their own customized best configuration in the binary map. On the other hand, when using the hybrid map, the equal weights among different criteria were hard-coded and inherited from the continuous map.

Finally, the participants on average preferred the binary map in overall sense. This might be attributed to the clear and easy-to-read distinction between the suitable and unsuitable working configurations by the binary states. Nevertheless, the differences for the preference were not statistically significant; therefore we recommend that subjective preference should be examined individually for a specific user.

## 5. Conclusion

In conclusion, we recommend that the selection of map should be primarily based on the different advantages of the maps with respect to the specific requirements of a given application. If maintaining the thresholds strictly is important, we recommend using the binary map. Subjective aspects can be considered as secondary reason for selection. For example, if easy-to-use aspect is important, we recommend the binary map. If higher resolution of states is require, the continuous map provides such intrinsic advantage. Nevertheless, in such case we recommend using the hybrid map instead of the pure continuous map, since it combines the advantages of the binary and continuous maps, at a slight expense of complexity compared to the binary map.

## Data Availability Statement

The original contributions presented in the study are included in the article/supplementary materials, further inquiries can be directed to the corresponding author/s.

## Ethics Statement

Ethical review and approval was not required for the study on human participants in accordance with the local legislation and institutional requirements. The patients/participants provided their written informed consent to participate in this study.

## Author Contributions

LP and CF developed the concept and methods. LP, DS, and CF contributed to programming, experiments and data analysis. LP wrote the first draft of the paper. LP and CF revised the paper. All authors read and approved the submitted version.

## Conflict of Interest

The authors declare that the research was conducted in the absence of any commercial or financial relationships that could be construed as a potential conflict of interest.
